# Phenotype correlations reveal the relationships of physiological systems underlying human ageing

**DOI:** 10.1111/acel.13519

**Published:** 2021-11-26

**Authors:** Meng Hao, Hui Zhang, Zixin Hu, Xiaoyan Jiang, Qi Song, Xi Wang, Jiucun Wang, Zuyun Liu, Xiaofeng Wang, Yi Li, Li Jin

**Affiliations:** ^1^ State Key Laboratory of Genetic Engineering Collaborative Innovation Center for Genetics and Development School of Life Sciences and Human Phenome Institute Fudan University Shanghai China; ^2^ National Clinical Research Center for Ageing and Medicine Huashan Hospital Fudan University Shanghai China; ^3^ Key Laboratory of Arrhythmias of the Ministry of Education of China Tongji University School of Medicine Shanghai China; ^4^ Research Unit of Dissecting the Population Genetics and Developing New Technologies for Treatment and Prevention of Skin Phenotypes and Dermatological Diseases (2019RU058) Chinese Academy of Medical Sciences Beijing China; ^5^ Center for Clinical Big Data and Analytics Second Affiliated Hospital and Department of Big Data in Health Science School of Public Health Zhejiang University School of Medicine Hangzhou Zhejiang China; ^6^ International Human Phenome Institutes Shanghai China

**Keywords:** composite phenotype analysis, functional ageing, human ageing, phenotypic ageing, physiological systems

## Abstract

Ageing is characterized by degeneration and loss of function across multiple physiological systems. To study the mechanisms and consequences of ageing, several metrics have been proposed in a hierarchical model, including biological, phenotypic and functional ageing. In particular, phenotypic ageing and interconnected changes in multiple physiological systems occur in all ageing individuals over time. Recently, phenotypic age, a new ageing measure, was proposed to capture morbidity and mortality risk across diverse subpopulations in US cohort studies. Although phenotypic age has been widely used, it may overlook the complex relationships among phenotypic biomarkers. Considering the correlation structure of these phenotypic biomarkers, we proposed a composite phenotype analysis (CPA) strategy to analyse 71 biomarkers from 2074 individuals in the Rugao Longitudinal Ageing Study. CPA grouped these biomarkers into 18 composite phenotypes according to their internal correlation, and these composite phenotypes were mostly consistent with prior findings. In addition, compared with prior findings, this strategy exhibited some different yet important implications. For example, the indicators of kidney and cardiovascular functions were tightly connected, implying internal interactions. The composite phenotypes were further verified through associations with functional metrics of ageing, including disability, depression, cognitive function and frailty. Compared to age alone, these composite phenotypes had better predictive performances for functional metrics of ageing. In summary, CPA could reveal the hidden relationships of physiological systems and identify the links between physiological systems and functional ageing metrics, thereby providing novel insights into potential mechanisms underlying human ageing.

## Graphical abstract

We proposed a composite phenotype analysis (CPA) strategy to analyse 71 biomarkers from 2074 individuals in the Rugao Longitudinal Ageing Study. CPA grouped these biomarkers into 18 composite phenotypes according to their internal correlation. These composite phenotypes could reveal the hidden relationships of physiological systems and identify the links between physiological systems and functional ageing metrics, thereby providing novel insights into potential mechanisms underlying human ageing.

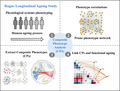

## 1 INTRODUCTION

Ageing is characterized by degeneration and loss of function across multiple physiological systems, and it has recently been investigated as a complex, multifactorial process (Maguire & Slater, 2013). To study the mechanisms and consequences of ageing, several metrics have been proposed in hierarchical models (Ferrucci et al., 2018), namely biological, phenotypic and functional ageing. In particular, phenotypic ageing and interconnected changes in multiple physiological systems occur in all ageing individuals over time and may contribute to clinical diseases (Ferrucci et al., 2018). For example, ageing is accompanied by a progressive decline in immune function, and toll‐like receptor 5 (TLR5) may provide a critical mechanism to enhance immune responsiveness in older individuals (Qian et al., 2012). Recently, researchers have (Liu et al., 2018) proposed a new ageing measure, phenotypic age, to capture morbidity and mortality risk across diverse subpopulations in a US cohort study (Levine et al., 2018). Although phenotypic age can facilitate the identification of individuals at risk for a number of health conditions and deaths, and although it may serve as a useful tool for evaluating intervention effectiveness (Belsky et al., 2015; Liu et al., 2018), it still has some drawbacks. Phenotypic age was calculated based on a linear combination of chronological age and 9 multisystem clinical chemistry biomarkers, which may overlook the relationships among physiological systems (Zierer et al., 2015). Meanwhile, multiple physiological systems play important but different roles in the ageing process (López‐Otín et al., 2013); thus, it was essential to explore the relationships between physiological systems.

To explore the relationships of physiological systems, systematically dissecting the correlation structure of these phenotypic biomarkers is essential. Analysis of the correlation structure of phenotypic biomarkers underlying human ageing may be a promising strategy (Freund, 2019). Generally, there are several methods of dissecting correlation structures from multiple phenotypic biomarkers, such as network‐based methods (Newman et al., 2012; Zierer et al., 2015) and composite phenotype‐based methods (Li et al., 2021). In network‐based methods, phenotypic biomarkers are mostly grouped into several physiological modules based on prior knowledge (Freund, 2019; Newman et al., 2012). However, in composite phenotype‐based methods, phenotypic biomarkers were grouped into several composite phenotypes using data‐driven approaches. One of the important strengths of composite phenotypes is their ability to reduce the data dimensions and capture efficient information from multiple single phenotypes (Li et al., 2021). In addition, the correlation between composite phenotypes can reflect the relationships of physiological systems. Therefore, composite phenotype analysis could be an efficient strategy to understand the relationships of physiological systems underlying human ageing.

Here, we proposed a new framework of composite phenotype analysis (CPA) and applied it to 71 phenotypic biomarkers in the Rugao Longitudinal Ageing Study (RLAS) to reveal the relationships of physiological systems underlying human ageing. First, we grouped phenotypic biomarkers into several composite phenotypes and examined the robustness of the clustering results. Second, we compared the correlation structure of composite phenotypes with prior findings to validate the reliability and investigate the potential mechanisms of correlations in these composite phenotypes. Finally, we linked composite phenotypes to functional metrics of human ageing, including disability, depression, cognitive function and frailty, and further validated the application of CPA in both cross‐sectional and longitudinal datasets.

## 2 RESULTS

### 2.1 Exploring the correlation structure of phenotypic biomarkers underlying human ageing

In this study, we collected 71 phenotypic biomarkers from 2074 individuals (44.94% males) from the fourth wave of the RLAS (Liu et al., 2016). The basic descriptive statistics are summarized in Table S1. Due to a lack of information on disability, cognitive impairment and frailty, 251 individuals were excluded from further analysis. The association of composite phenotypes with functional ageing metrics was analysed in 1823 individuals. Among them, 799 (43.83%) were males, and the mean age was 78.68 ± 4.79 years. According to the criteria, 169 (9.27%), 909 (49.86%) and 322 (17.66%) individuals were defined as having disability, cognitive impairment and frailty, respectively. The health status of the individuals was described in Table S2.

**FIGURE 1:**
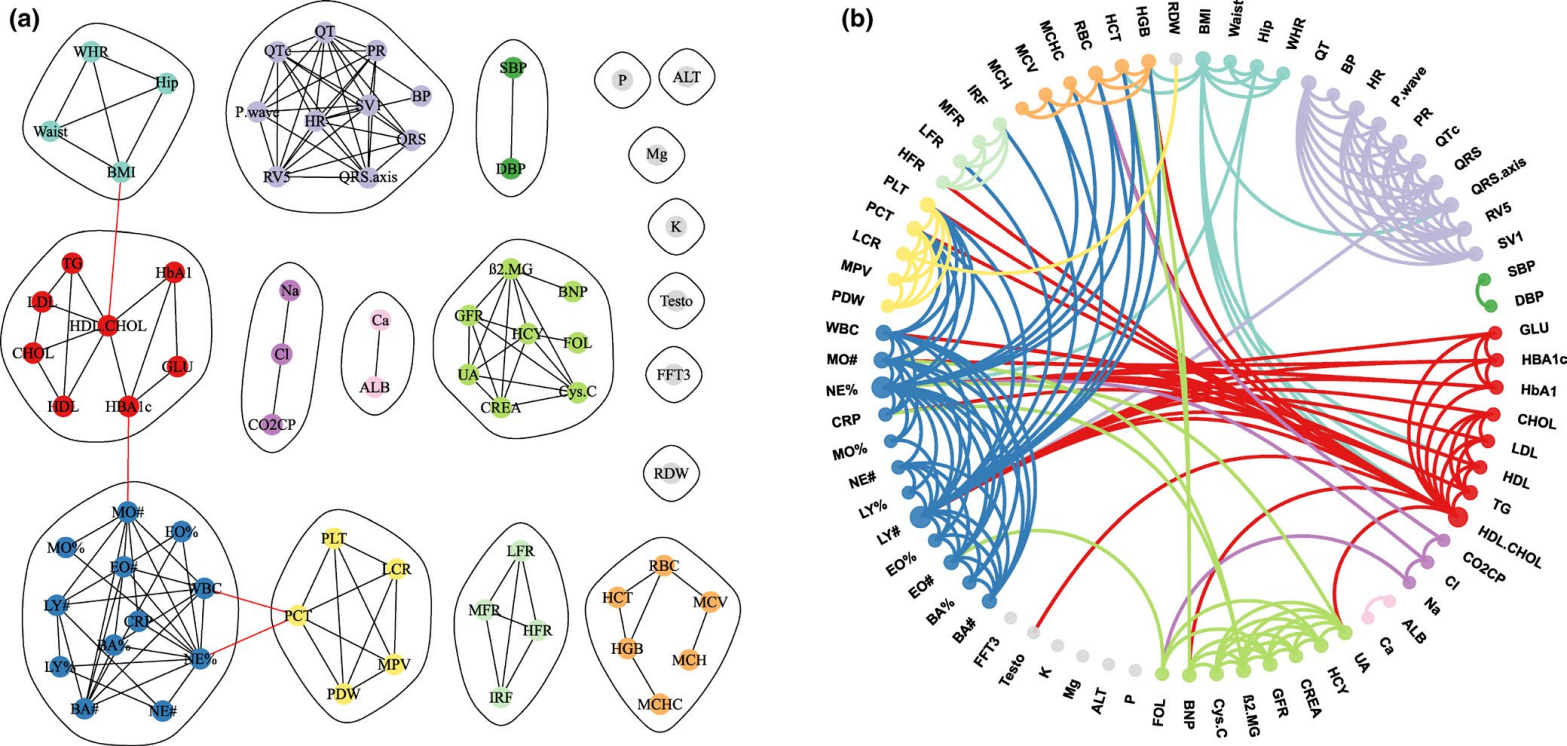
Sparse phenotypic network of 71 biomarkers after filtering. The threshold was set as 0.16 (a) and 0.14 (b). The 18 clusters are marked by different colours, while the isolated phenotypes are marked in grey

We used the maximum information coefficient (MIC) (Reshef et al., 2011) to measure both linear and nonlinear correlations between phenotypic biomarkers. To detect and filter spurious correlations in high‐dimensional phenotypic datasets, we applied random matrix theory (Luo et al., 2007) to identify the appropriate threshold of MIC. We found that the eigenvalue spacing distribution of the MIC correlation matrix transitioned from a Winger–Dyson distribution to an exponential distribution when the candidate signal‐noise separating threshold was set as 0.16 (Figure S1). The elements in the MIC correlation matrix which were less than the threshold were set as zero (Figure S2). The filtered MIC correlation matrix was then used as the adjacency matrix of the phenotypic network. Next, a sparse phenotypic network was built with each phenotypic biomarker as a vertex and filtered MIC as a weighted edge (Figure 1a). Furthermore, to detect communities of these biomarkers, spectral clustering was applied on the sparse phenotypic network.

To examine the robustness of the clustering results, the samples were initially resampled 100 times, and the present clustering results were used as a reference. Then, normalized mutual information (NMI) was applied to quantify the concordance between the resampled results and the reference. The mean NMI was over 0.985, indicating that the clustering results were robust for resampled samples (Figure S3A). To further evaluate the impact of thresholds on clustering results, the threshold was set from 0.1 to 0.5. When the threshold was set to approximately 0.16, the clustering results were stable compared with the reference (Figure S3B). Furthermore, we also examined the impact of the threshold on the topology of the phenotypic network (Figure S3C). The topological parameters included connectance, average path length, clustering coefficient and modularity. The distribution of connectance showed that the phenotypic network became sparse with the strict threshold. Meanwhile, the average path length, clustering coefficient and modularity reached the optimized peak value. These results indicated that the phenotypic structure was highly modularized with a threshold set to approximately 0.16.

In summary, we used MIC to quantify the correlations among phenotypic biomarkers and filtered the spurious correlation with the threshold suggested by random matrix theory. The structures of phenotypic biomarkers were identified through spectral clustering. In addition, the clustering results were robust to samples, and the threshold setting was reasonable. The relationships within clusters were much stronger than those between clusters (Figure S2). The correlation heat map (Figure S2) showed the spectral clustering results of 71 phenotypic biomarkers, which were clustered into 18 groups.

### 2.2 Defining composite phenotypes of phenotypic biomarkers underlying human ageing

Based on the above clustering results of 71 biomarkers, we obtained 18 clusters and defined each cluster as a composite phenotype (CP). Overall, 18 composite phenotypes (CPs) were extracted, and we defined the composite phenotypes by clinical implications based on their contained single phenotypes (Table 1, Figure 1a). The composite phenotypes include body shape (CP1), electrocardiography (CP2), blood pressure (CP3), blood lipids and glucose (CP4), blood gas and electrolytes (CP5), liver function and electrolytes (CP6), kidney and cardiovascular functions (CP7), electrolytes (CP8, CP10, CP11), liver function (CP9), hormones (CP12, CP13), white blood cells (CP14), platelets (CP15), reticulocytes (CP16), red blood cell counts (CP17) and red blood cell distribution (CP18). Most of the composite phenotypes were highly consistent with prior knowledge. For example, CP1 included several physical phenotypes of body shape, such as BMI and WHR. CP18 included platelet‐related phenotypes (MPV, PCT, PDW, LCR, PLT). In addition, compared with prior knowledge, composite phenotypes could provide additional clues to explore the intrinsic relationships of these phenotypes. CP4 included phenotypes of blood lipids (TG, HDL, CHOL, LDL) and phenotypes of blood glucose (GLU, HBA1c, HbA1). Glucose and lipid metabolism are linked through complex interactions, clinically manifesting as diabetic dyslipidaemia (Parhofer, 2015). CP5 included electrolytes (Na, Cl) and blood gas (CO2CP). The transport of carbon dioxide is dependent on a chloride shift, which refers to the exchange of bicarbonate and chloride across the membrane of red blood cells (Crandall et al., 1981). The CP7 included indicators of kidney function (CREA, eGFR, UA, β2. MG, Cys. C) and cardiovascular function (HCY, FOL, BNP). Several epidemiological studies have confirmed the relationships between chronic kidney disease and cardiovascular risk factors (Amann et al., 2006). CP14 grouped CRP into white blood cell phenotypes, both of which are indicators of inflammation. Therefore, these results indicated that CPA could reveal novel and meaningful classifications of these phenotypic biomarkers underlying human ageing.

**TABLE 1:** Details of 18 composite phenotypes

Composite phenotype	Single Phenotype	Clinical implications
CP1	BMI, Waist, Hip, WHR	Body shape
CP2	BP, QT, HR, P.wave, QTc, QRS.axis, PR, RV5, SV1, QRS	Electrocardiography
CP3	SBP, DBP	Blood pressure
CP4	TG, HDL.CHOL, HBA1c, GLU, HbA1, HDL, LDL, CHOL	Blood lipids, blood glucose
CP5	CO2CP, Cl, Na	Blood gas/CO2CP, electrolytes/ Cl, Na
CP6	ALB, Ca	Liver/ALB, electrolytes/Ca
CP7	HCY, CREA, eGFR, UA, β2.MG, Cys.C, FOL, BNP	Kidney, cardiovascular
CP8	P	Electrolytes/P
CP9	ALT	Liver/ALT
CP10	Mg	Electrolytes/Mg
CP11	K	Electrolytes/K
CP12	Testo	Hormone/Testo
CP13	FFT3	Hormone/FFT3
CP14	BA#, BA%, EO#, EO%, LY#, MO#, NE%, WBC, LY%, NE#, MO%, CRP	White blood cell
CP15	MPV, PCT, PDW, LCR, PLT	Platelet
CP16	HFR, MFR, LFR, IRF	Reticulocyte
CP17	RBC, HCT, HGB, MCV, MCHC, MCH	Red blood cell/Count
CP18	RDW	Red blood cell/Distribution

The correlations of individual phenotypic biomarkers within the same composite phenotypes were primarily identified by the sparse phenotypic network (Figure 1a). In addition, the relationships among single phenotypic biomarkers across different composite phenotypes were investigated in the circular phenotypic network with a relatively low threshold (Figure 1b). The correlations between individual phenotypes with MICs greater than 0.1 are supplemented in Table S3. In particular, phenotypes of body shape (CP1), blood lipids (CP4) and white blood cells (CP14) were significantly connected. Additionally, the phenotypes of kidney and cardiovascular functions (CP7), white blood cells (CP14) and red blood cell counts (CP17) were significantly connected.

### 2.3 Linking composite phenotypes and functional ageing metrics

**FIGURE 2:**
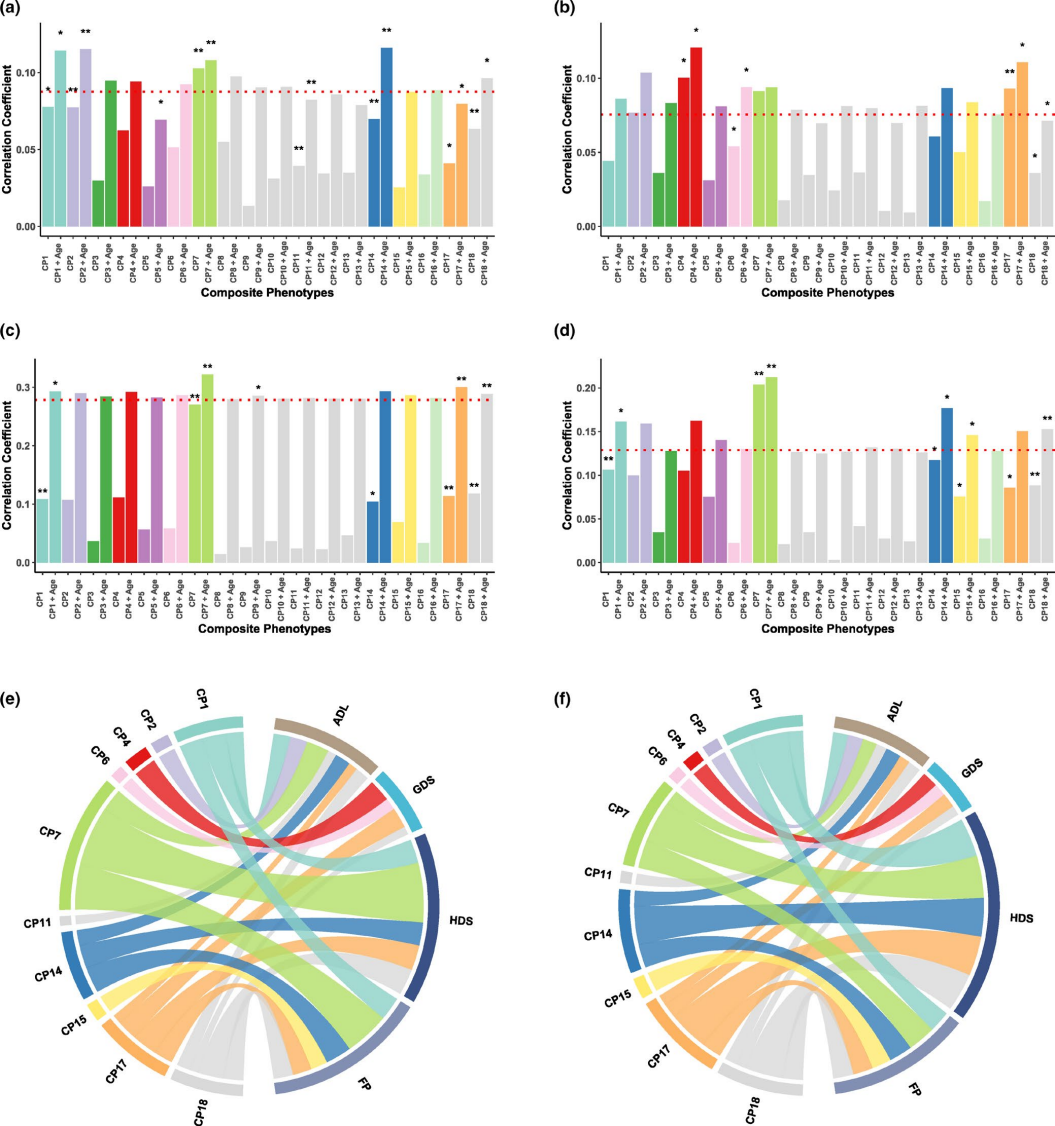
Histograms of the correlation between composite phenotypes, age and functional ageing metrics (a‐d). The correlation coefficients between age and functional ageing metrics (a: ADL, b: GDS, c: HDS, d: FP) are marked by red dotted lines (Model 2). Each composite phenotype has two same coloured histograms (left: Model 1, right: Model3). The asterisk (*) indicates the significance of association between composite phenotypes and functional ageing metrics. The chord plots of the correlation between composite phenotypes, age and functional ageing metrics (e, f). The left panel shows the correlations of Model 1 (e), and the right panel shows the correlations of Model 3 (f)

To investigate the ageing signatures of composite phenotypes, we linked them with functional ageing metrics **(**Figure 2), including disability (ADL) (Katz et al., 1970), depression (GDS) (Dennis et al., 2012), cognitive function (HDS) (Imai & Hasegawa, 1994) and frailty (FP) (Fried et al., 2001). First, we used multiple linear regression to investigate the associations between composite phenotypes and functional ageing metrics (Model 1). Then, these associations were compared with those between age and the functional ageing metrics (Model 2). Finally, the combinations of composite phenotypes and age were linked to functional ageing metrics to explore the additional ageing signatures of composite phenotypes except for age (Model 3). These associations between composite phenotypes and functional ageing metrics were adjusted for covariates including marital status and educational levels (Model 4). These models were conducted in males and females separately (Table S4). Similar results were found in males and females.

For disability (ADL, Figure 2a), we found that CP7 were more informative than age in predicting disability. CP1, CP2, CP11, CP14, CP17 and CP18 had additional effects with age to correlate with disability. For depression (GDS, Figure 2b), we found that CP4 and CP17 were more informative than age. CP6 and CP18 had additional effects with age that correlate with depression. For cognitive function (HDS, Figure 2c), there were no composite phenotypes that outperformed age. However, CP1, CP7, CP17 and CP18 had additional effects with age to correlate with cognitive function. For frailty (FP, Figure 2d), CP7 was more informative than age. In addition, CP1, CP14, CP15 and CP18 had additional effects with age to correlate with frailty.

In summary, we found that body shape (CP1), kidney and cardiovascular functions (CP7), white blood cells (CP14) and red blood cell counts (CP17) and distribution (CP18) were primarily associated with functional ageing metrics (Figure 2e). Cognitive function had the best correlation with these composite phenotypes, followed by frailty, disability and depression. With the addition of age, there were few improvements in the correlation between the composite phenotype of body shape (CP1), kidney and cardiovascular functions (CP7) and functional ageing metrics. In contrast, several composite phenotypes (CP14, CP17, CP18) had great additional effects with age that correlate with functional ageing metrics (Figure 2f). After adjusting for covariates in Model 4, the composite phenotypes were still significantly correlated with functional ageing metrics (Table S4).

### 2.4 Replicating the associations between composite phenotypes and functional ageing metrics

**FIGURE 3:**
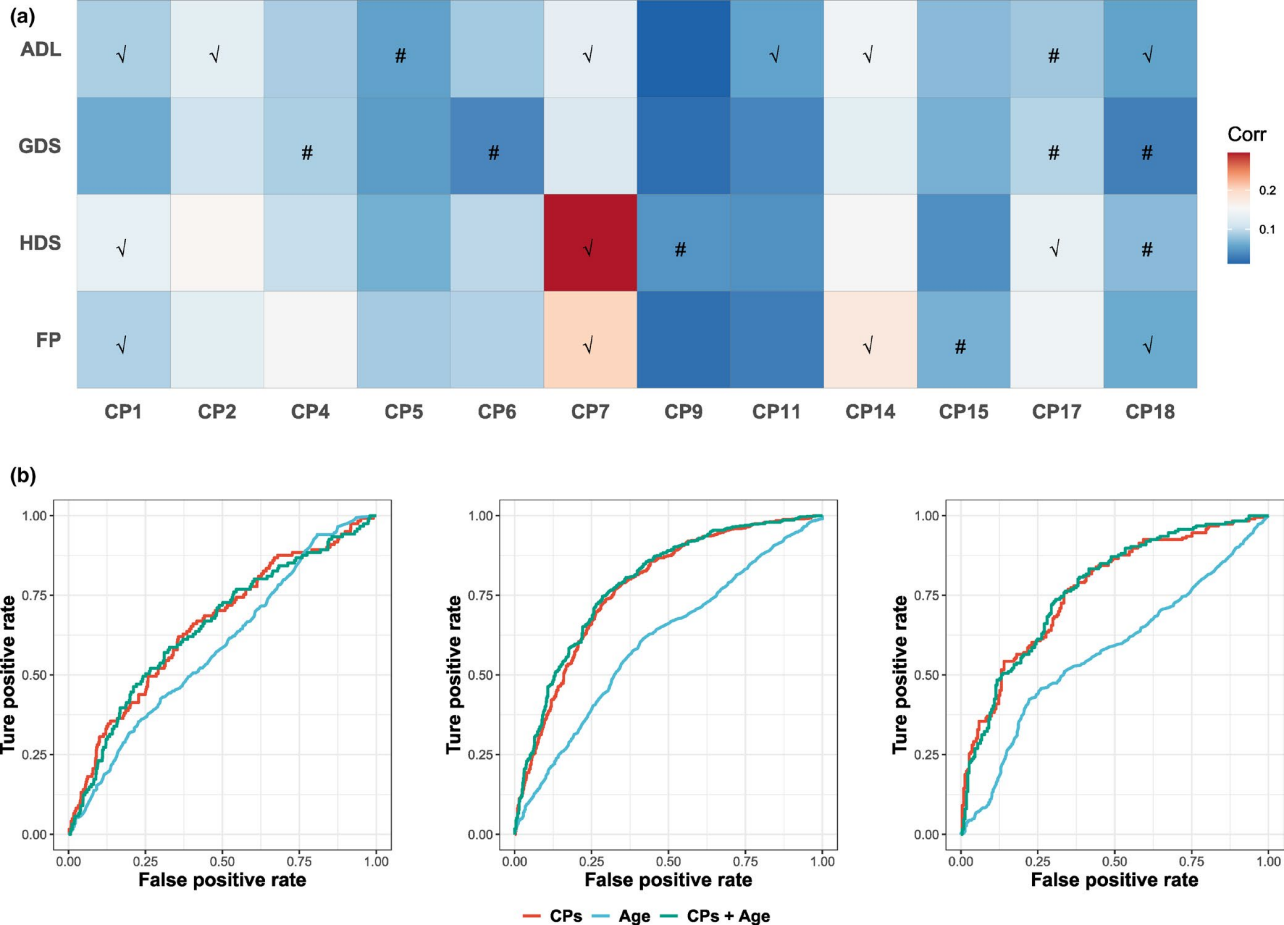
Heat map of the correlation between composite phenotypes and functional ageing metrics in the validation data (a). The heat map cell represents the correlation between composite phenotypes and functional ageing metrics in second and third waves of RLAS. The replicated correlations are marked with check mark (√) and not replicated are marked with number sign (#). ROC curves (b) of predictions for disability (left), cognitive function (middle) and frailty (right)

To validate the applications of CPA, we first examined these associations in other cross‐sectional data of RLAS (i.e. second and third waves). Most of the significant associations observed in the fourth wave of RLAS data were consistent with those in the validation data (Figure 3a). CP1 (body shape), CP7 (kidney and cardiovascular functions) and CP14 (white blood cells) had remarkable correlations with functional ageing metrics. However, the associations between depression and composite phenotypes were not replicated. The associations between counts and distribution of red blood cells (CP17, CP18) and functional ageing metrics were partially validated.

Furthermore, we predicted 3‐year disability, cognitive function and frailty in the fourth wave of data (2019) based on second‐wave data (2016) from the RLAS. Receiver operating characteristic (ROC) curves were used to quantify the performance of the prediction (Table 2, Figure 3b). The area under the curve (AUC) of age for the three functional ageing metrics was approximately 0.6. For all composite phenotypes, the AUC reached 0.656 for disability, 0.777 for cognitive decline and 0.773 for frailty. The addition of age failed to improve the performance of the models. These findings implied that the ageing information underlying age overlapped in composite phenotypes. For composite phenotypes, CP1 (body shape), CP7 (kidney and cardiovascular functions) and CP17 (red blood cell counts) had remarkable AUCs for functional ageing metrics.

**TABLE 2:** AUCs of composite phenotypes and age for functional ageing metrics

Composite Phenotype	ADL	HDS	FP
CPs	0.656	0.777	0.773
Age	0.581	0.608	0.600
CPs + Age	0.663	0.792	0.780
CP1	0.617	0.752	0.702
CP2	0.550	0.643	0.592
CP4	0.510	0.571	0.566
CP5	0.507	0.518	0.560
CP6	0.548	0.562	0.585
CP7	0.614	0.769	0.715
CP9	0.550	0.542	0.526
CP11	0.564	0.538	0.546
CP14	0.554	0.562	0.572
CP15	0.574	0.587	0.579
CP17	0.521	0.675	0.705
CP18	0.548	0.571	0.608

### 2.5 Revealing relationships between kidney and cardiovascular functions in CP7

**FIGURE 4:**
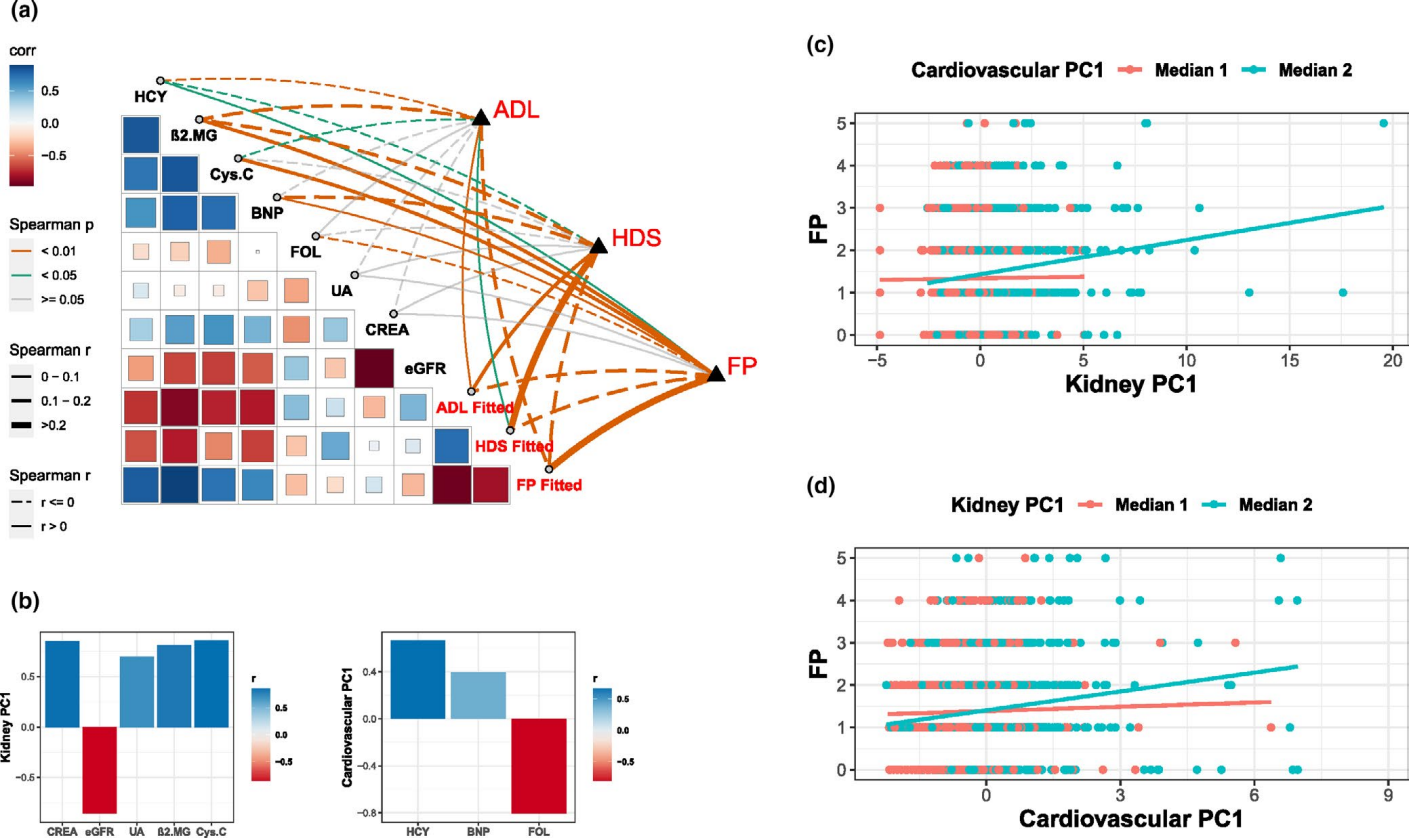
Combined plots of CP7 (a). The bottom left heat map shows the correlation between single phenotypes of CP7. The top right plots show the correlations between phenotypes and functional ageing metrics. Histograms of correlations (b) between PCs and single phenotypes. Scatter plots of frailty, kidney PC1 (c) and cardiovascular PC1 (d). The linear regressions are marked with different coloured lines

Considering the great correlation between CP7 and functional ageing metrics, we investigated the advantages of composite phenotypes using CP7. Compared with single phenotypes, one of the most important strengths of composite phenotypes was that they could capture the information of multiple single phenotypes, thus suggesting shared mechanisms. The individual phenotypes of CP7 included kidney function indicators (CREA, eGFR, UA, β2.MG, Cys.C) and cardiovascular indicators (BNP, HCY, FOL). There were strong correlations among them, and they were grouped as one composite phenotype (Figure 4a). With three functional ageing metrics (disability, cognitive function and frailty), we first fitted individual phenotypes of CP7 to the metrics with multivariable linear regressions separately. The composite phenotype was defined as a set of individual phenotypes, and CP7 retained all information of these individual phenotypes, outperforming individual phenotypes in correlating with functional ageing metrics. These findings comprehensively suggested a decline in kidney and cardiovascular functions with human ageing.

Furthermore, we explored the interactions between the kidney and cardiovascular functions using these biomarkers in CP7. Notably, we found that kidney and cardiovascular functions (CP7) were associated with frailty and that the interactions between them on frailty were also significant (Figure 4c,d). Specifically, we conducted principal component analysis on these indicators (Figure 4b). The first principal component (PC1) was used as the primary feature for kidney and cardiovascular functions. For five kidney function indicators, kidney PC1 explained 67% of the variance, and a higher value of PC1 suggested impaired kidney function. For the three cardiovascular function indicators, cardiovascular PC1 explained 41% of the variance, and a higher value of PC1 suggested impaired cardiovascular function. Then, we explored the interaction of kidney PC1 and cardiovascular PC1 on frailty. Kidney PC1 was positively correlated with FP score, indicating a decline in kidney function with frailty. Its effects on frailty increased with higher cardiovascular PC1 levels (Figure 4c). Similarly, cardiovascular PC1 was also a risk factor for frailty, and its effects on frailty increased with higher kidney PC1 levels (Figure 4d). In summary, there were nonlinear additional effects of kidney and cardiovascular functions on frailty, suggesting synergistic effects between kidney and cardiovascular functions underlying human ageing.

### 2.6 Evaluating the performance of composite phenotype analysis (CPA)

**FIGURE 5:**
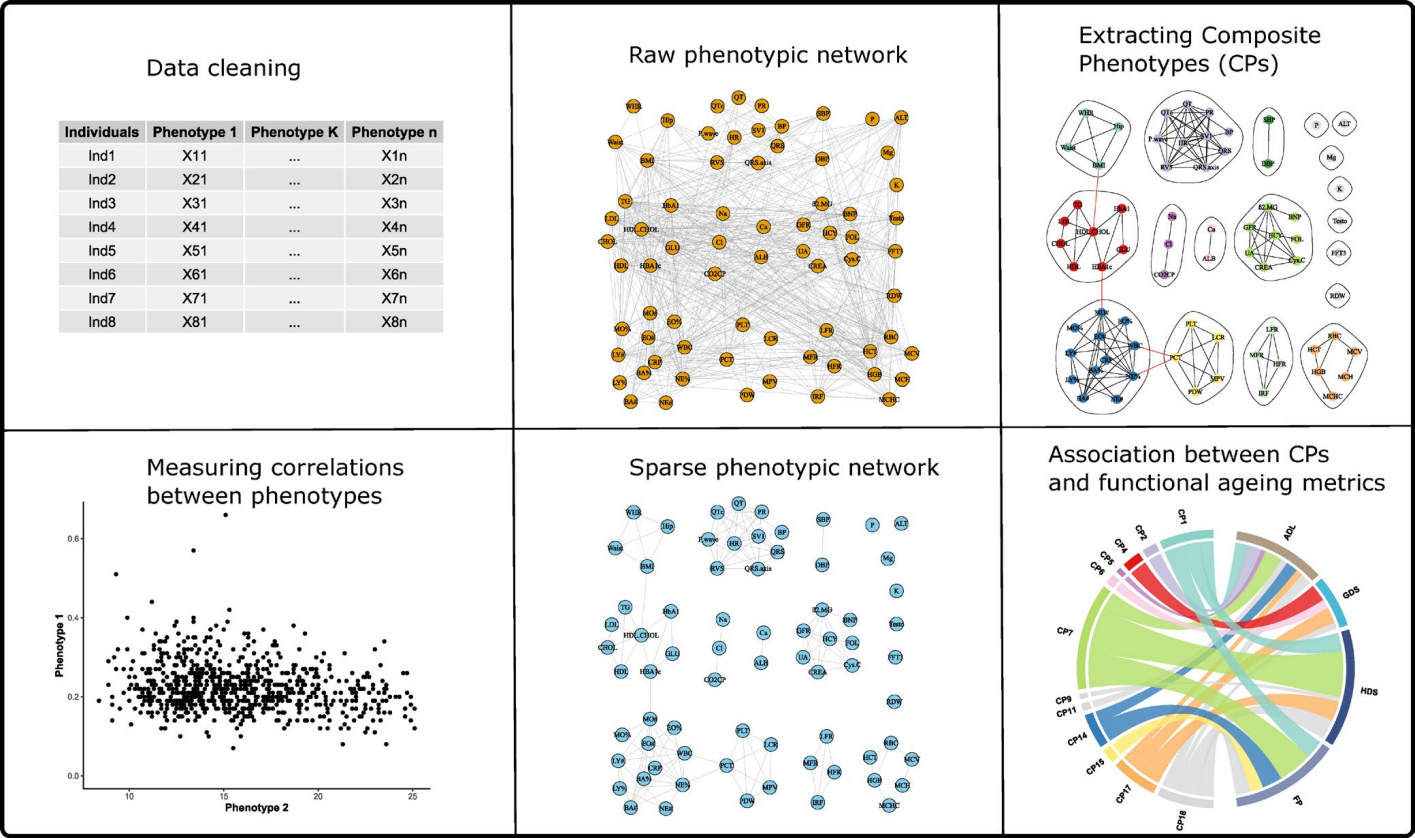
The workflow of composite phenotype analysis

We proposed CPA as an integrated framework to systematically dissect the phenotype correlations in the RLAS. The workflow is summarized in Figure 5 and mainly consists of four steps: measuring the correlation between phenotypes (Step 1), pruning the phenotypic network (Step 2), extracting the composite phenotypes (Step 3) and linking the composite phenotypes to functional ageing metrics (Step 4). To investigate the performance of CPA, other three correlation measurements, three filtering thresholds, five clustering algorithms and five dimensionality reduction methods were enrolled. The benchmarking analysis was separated into four parts (Experimental procedures).

In summary, the advantages of CPA could be seen in several ways. First, both linear and nonlinear correlations between phenotypes were considered. Then, spectral clustering grouped the phenotypes based on Laplacian matrices of the phenotypic network. The sparsity of the network contributed to the performance of spectral clustering, while other clustering algorithms were subject to the noise of spurious correlations between phenotypes. Finally, the composite phenotypes retained all the information of individual phenotypes. Linear regression and machine learning algorithms were applied to explore the connection between phenotypic and functional ageing. In CPA, we found that CP1, CP7, CP14, CP17 and CP18 had remarkable correlations and predictive abilities with functional ageing metrics.

## 3 DISCUSSION

In this study, we applied CPA to 71 biomarkers underlying human ageing to elucidate their correlation structure and obtained 18 composite phenotypes in the RLAS. These composite phenotypes captured more ageing information than age in correlation with functional ageing metrics, including disability, depression, cognitive function and frailty. In particular, CP1 (body shape), CP7 (kidney and cardiovascular functions), CP14 (white blood cells), CP17 (red blood cell counts) and CP18 (red blood cell distribution) had remarkable correlations and predictive abilities with functional ageing metrics. Additionally, we found a significant correlation between blood lipids and blood glucose (CP4), a correlation between electrolytes and blood gas (CP5), and a correlation between kidney and cardiovascular functions (CP7). Furthermore, the effects of these phenotypic biomarkers on functional ageing metrics were not independent (as with CP7). In brief, there were interactions between these biomarkers, suggesting extensive relationships between these physiological systems underlying human ageing.

The potential mechanisms of correlations between composite phenotypes and functional ageing metrics were reasonable. Both CP1 (body shape) and CP14 (white blood cells) were associated with functional ageing metrics in our study. The potential mechanisms underlying this association may be that low‐grade systemic inflammation was associated with CP1 (Visser et al., 1999) and CP14 (Leng et al., 2005). Inflammation may mediate the association of CP1 and CP14 with frailty (Leng et al., 2009), cognitive impairments (Zenaro et al., 2015) and disability (Nuesch et al., 2012) in older adults. CP17 (red blood cell counts) and CP18 (red blood cell distribution) were associated with cognitive impairments in our study. Previous studies observed similar results; mean cell haemoglobin (MCH) and red cell distribution width (RDW) were most strongly associated with cognitive function (Winchester et al., 2018) and iron deficiency anaemia (Goddard et al., 2011). Mendelian randomization studies demonstrated that increased iron reduces the risk of Parkinson's disease (Pichler et al., 2013). In addition, low‐grade inflammation was associated with the development of anaemia (Nemeth & Ganz, 2014) and frailty (Soysal et al., 2016) in older adults; therefore, this could be one of the potential pathways underlying the association between anaemia and frailty.

The correlation structures between phenotypes in the RLAS, such as blood glucose and blood lipids (CP4), kidney and cardiovascular functions (CP7), are worthy of further investigation. The health of the population could contribute to the observed correlation structures. Therefore, we compared the correlations between phenotypes within healthy individuals and individuals with diseases (e.g. cardiovascular disease, chronic kidney disease and anaemia). The correlations between indicators of kidney and cardiovascular functions were enhanced in subgroups with diseases (Figure S4). For example, the SCC between β2. MG and BNP was 0.44, while it was 0.27 in the healthy subgroups. The dysregulation of physiological systems could lead to distinctive phenotype correlations, which was more evident in diseases.

Relationships among physiological systems are common, such as cardiopulmonary and brain–heart systems (Kuh et al., 2019; Schefold et al., 2016). Here, we also explored the interactions between the kidney and cardiovascular functions. The mechanisms by which the kidney and cardiovascular system are associated with frailty are not entirely understood. Probable explanations of the interaction were that cardiac and renal disease share several common bidirectional pathways, such as haemodynamic, (neuro) hormonal and cardiovascular disease‐associated mechanisms (Schefold et al., 2016). All three mechanisms are interconnected and could negatively affect both cardiac and renal function (Schefold et al., 2016), thereby causing frailty by influencing physical and cognitive function.

Statistical approaches to infer networks from biological data include Gaussian graph models, Bayesian networks, correlation networks and information theory (Yu et al., 2013). It is essential to choose an appropriate method to quantify the similarity between the vertices of the network. We used the maximal information coefficient (MIC) to detect the correlations between phenotypes, which serves as a general tool in coexpression networks (Song et al., 2015). With the increasing availability of biological data, filtering information in large complex networks of interactions is beneficial for the emergence of biological networks (Marcaccioli & Livan, 2019). In this study, we applied a global threshold developed from RMT (Luo et al., 2006). The thresholding methodology of RMT has been applied in gene and microbial networks (Deng et al., 2012; Luo et al., 2007). The suitability of the threshold was evaluated through its effects on the topology of the networks (Couto et al., 2017). RMT has been widely used in characterizing nonrandom phenomena in physical, material and social systems (Luo et al., 2007), and it has been well recognized in these systems that RMT analyses are efficient for distinguishing system‐specific, nonrandom properties from random noise (Luo et al., 2006; Segal et al., 2003).

Network‐based methods have been widely used in many fields, such as microbial communities, protein interactions and gene coexpression. In the framework of network‐based methods, phenotypic biomarkers were mostly grouped into several physiological modules based on prior knowledge (Freund, 2019; Newman et al., 2012). However, in CPA, phenotypic biomarkers were grouped into several composite phenotypes using data‐driven methods. Therefore, we proposed using CPA to study ageing for the first time and revealed several relationships of physiological systems underlying human ageing. CPA could potentially be employed as a general strategy for studying complex traits, especially in the analysis of phenomics (Li et al., 2021).

There were still some limitations in our study. First, we enrolled 71 markers to construct the phenotypic network and extract composite phenotypes. This may differ in other cohorts and change with different phenotypes or approaches. Second, the number of biomarkers in our cohort increased with each of the three waves, although some biomarkers were not measured in the previous waves. Finally, although the study cohort had been followed up for three years and found encouraging results, it was still essential to conduct longer‐term follow‐ups to validate our findings.

In summary, CPA provides a promising opportunity for researchers to understand the intrinsic correlation structure of phenotypic ageing biomarkers through a data‐driven strategy. Furthermore, CPA could reveal the hidden relationships of physiological systems and identify the important links between physiological systems and functional ageing metrics, thereby providing novel insights into potential mechanisms underlying human ageing.

## 4 EXPERIMENTAL PROCEDURES

### 4.1 Study population

We applied CPA to data from the fourth wave of the ageing arm of the Rugao Longitudinal Ageing Study (RLAS), a population‐based observational two‐arm cohort study conducted in Rugao, Jiangsu Province, China (Liu et al., 2016). The validation data were drawn from the second wave of the cohort. As previously described, the second‐wave examination was conducted in April 2016 (wave 2), and the fourth‐wave examination was conducted in November 2019 (wave 4). Demographic, clinical, laboratory and anthropometric characteristics were collected from the RLAS. Demographic data included age, sex, marital status and educational years. Clinical characteristics, including cardiovascular disease (CVD), hypertension, chronic lung disease, cognitive function decline (assessed by the revised Hasegawa's dementia scale), antihypertension drugs and antidiabetic drugs. CVD included stroke, myocardial infarction and heart failure.

### 4.2 Biomarker datasets

Fasting blood samples of all participants were collected by trained nurses during the morning of the survey. Laboratory measurements (Table S1) included blood biochemistry (e.g. blood lipids), routine clinical examinations (e.g. blood pressure) and other blood biomarkers (e.g. homocysteine and B‐type natriuretic peptide). Anthropometrics characteristics were measured. Grip strength was assessed using a Hand Grip Dynamometer (Shanghai Wanqing Rlrctron Co. Ltd., Shanghai, China), timed ‘up and go’ test (participants stand up from an armchair, walk 3 m, return and sit down again), 5‐metres walking test and sit‐to‐stand from a chair test (Podsiadlo & Richardson, 1991). Electrocardiography was performed on each participant, and the ECG parameters, including heart rate, PR intervals (the time elapsing between the beginning of the P wave and the beginning of the next QRS complex), QRS duration (a series of waveforms on an electrocardiogram that represents depolarization of ventricular muscle cells), S wave in V1 (SV1), R wave in V5 (RV5) and QTc, were determined via the interpretation programs of the ECG machine. All ECG parameters and abnormalities were identified by another cardiologist. To adjust for heart rate, the Bazett formula (QTc =QT/√RR) was used in the present study.

### 4.3 Functional metrics of ageing

Functional disability was assessed by the Katz scale (Katz et al., 1970). Each task had three response options: strongly independent, somewhat independent and strongly dependent. Participants who responded as somewhat independent or strongly dependent for any tasks were defined as having a functional disability. Depressive symptoms were measured using the 15‐item Geriatric Depression Scale (GDS) (Yesavage, 1988), a validated self‐report questionnaire commonly used for the assessment of depressive symptoms in older adults. The questionnaire contained 15 questions (yes or no) with a score of 0–15. In our study, a score of 6 or more was defined as having a depressive symptom (Dennis et al., 2012). Cognitive function was evaluated by the revised Hasegawa's dementia scale (HDS‐R), which comprised orientation, memory, attention/calculation and verbal fluency (Imai & Hasegawa, 1994). HDS‐R has been widely accepted in Asian populations in clinical and epidemiological surveys for the assessment of cognitive impairment (Sengchanh et al., 2019). In our study, individuals who scored higher than 21.5 were defined as having normal cognitive function, while those who scored 21.5 or below were defined as having cognitive impairment. According to Fried et al., the frailty phenotype was defined in the following five domains: weight loss, exhaustion, low activity, weakness and slowness (Fried et al., 2001).

### 4.4 Composite Phenotype Analysis (CPA)

The correlation between phenotypes was quantified by MIC (Reshef et al., 2011). The idea of MIC was that a scatterplot of the two variables could be partitioned to encapsulate the relationship. It explored all grids up to a maximal grid resolution to obtain the highest normalized mutual information between these two variables. The calculation of MIC was implemented with the R package ‘Minerva’. We calculated the MIC in males and females separately and averaged the results. The thresholds of the MIC correlation matrix were obtained through RMT (Luo et al., 2007). In detail, the nearest‐neighbour spacing distribution (NNSD) for the eigenvalues of a random symmetric matrix followed the Wigner–Dyson distribution. While there were only strong correlations along the (block) diagonal of the matrix, it followed an exponential distribution. Therefore, with increasing threshold, the NNSD of the matrix transitioned from a Wigner–Dyson distribution to an exponential distribution. The R package ‘RMThreshold’ provided algorithms based on RMT that could be used to determine an objective threshold for signal‐noise separation in large random matrices. Community detection of phenotypic networks used spectral clustering algorithms. The topological parameters of the network included the average path length, clustering coefficient, connectance and modularity (Zhao & Liu,).

### 4.5 Statistical analysis of composite phenotypes and functional ageing metrics

The composite phenotypes were linked to functional ageing metrics using multiple linear regression. We constructed four models for each composite phenotype. First, we regressed the single phenotypes within the same composite phenotypes on functional ageing metrics, such as ADL. The Spearman correlation coefficients between the fitted value and observed value were calculated, and the F‐test was used to determine the significance of the model. The correlation coefficients were calculated in males and females separately, and p values of the F‐test were combined using Fisher methods. Second, we compared the correlation coefficients of Model 1 and Model 2. Finally, we used the likelihood‐ratio test (LRT) to assess the goodness of fit of Model 2 and Model 3. The significance of LRT indicated that composite phenotypes had additive effects with age. The covariates including educational levels and marital status were adjusted in Model 4. The LRT was used to test the significance of these associations adjusting for covariates. These models were conducted in males and females separately. The correlation coefficient and p values of the models are shown in Table S4. Machine learning models, including generalized linear models, support vector machines, random forests and elastic net regression, were used to calibrate the prediction of functional ageing metrics. Next, we used threefold cross‐validation to train these models and measure the performance of the model in test data using the R package ‘caret’. All analyses were performed in R V4.0.3.

Model 1: Y ~ CP (Single Phenotypes)
Model 2: Y ~ Age
Model 3: Y ~ CP + Age
Model 4: Y ~ CP + Covariates (Education + Marital status)

### 4.6 The Benchmarking of CPA

The evaluation of CPA was separated into 4 parts to compare its performance with other common algorithms.

At Step1, we compared MIC with the Spearman correlation coefficient (SCC), Pearson correlation coefficient (PCC) and Kendall correlation coefficient (KCC) and found that the MIC was proportional to these others (Figure S5A). We also found that some outliers were small on other coefficients but large on MIC, and the nonlinear relationships of these phenotypic biomarkers were quantified by MIC effectively. For example, the MIC between the ratio of waist to hip (WHR) and hip was 0.477, the SCC was 0.05, the PCC was 0.1 and the KCC was 0.027 (Figure S5A). These results indicated that MIC outperformed SCC, PCC and KCC in quantifying the nonlinear correlations between phenotypic biomarkers.

At Step2, the raw phenotypic network was pruned through thresholds to obtain the sparse network, in which the relationships of phenotypes were more distinct. We compared RMT with methods of multiple testing correction (Bonferroni adjustment and false discovery rate correction) and empirical thresholds. The distributions of the correlation coefficient were checked (Figure S5B). There were 1886 and 1222 remaining correlations for Bonferroni and false discovery rate (FDR), respectively, indicating that multiple testing corrections of p values to screen correlation were not enough. The top 5% and 10% of MIC were 0.139 and 0.215, respectively. According to the distribution of topology on the phenotypic network, these thresholds were less appropriate.

At Step3, a spectral clustering algorithm was used to extract composite phenotypes based on the sparse phenotypic network. Five other clustering methods, including K‐means clustering, partitioning around medoids (PAM), hierarchical clustering (Hclust), clustering large applications (CLARA) and divisive analysis clustering (DIANA), were also applied. The classification of phenotypes by CPA was consistent with prior knowledge and was used as a reference to evaluate the performance of other clustering algorithms. The results of other methods were aligned with the reference (Figure S5C). The performance of CLARA was the best, with an NMI equal to 0.734. Although it successfully captured the partial characteristics of composite phenotypes such as CP7, it was laborious to group the phenotypes and obtain reasonable classification.

At Step4, the composite phenotypes were linked to functional ageing metrics through linear regression. Other dimensionality reduction methods, including principal component analysis (PCA), canonical correlation analysis (CCA), partial least squares regression (PLS), nonnegative matrix factorization (NMF) and locally linear embedding (LLE), were also applied to construct the correlation between the composite phenotype and functional ageing metrics (Figure S5D). The single phenotypes in CP7 were taken as an example in investigating the performance of these methods. CP7, which was defined as a set of single phenotypes rather than a fixed latent variable, had the strongest correlation with these metrics. The components extracted by PLS, CCA and PCA were significantly correlated with disability, cognitive impairments and frailty. However, dimension reduction impaired the correlation compared with CPA.

## CONFLICT OF INTEREST

The authors declare that they have no competing interests.

## AUTHOR CONTRIBUTIONS

Conceptualization: Li Jin, Xiaofeng Wang, Yi Li, Zuyun Liu. Sample collection: Xiaofeng Wang, Xiaoyan Jiang, Hui Zhang, Qi Song, Xi Wang, Jiucun Wang. Data curation: Hui Zhang, Xiaofeng Wang. Investigation: Meng Hao, Hui Zhang, Yi Li, Zixin Hu. Methodology: Meng Hao, Yi Li, Hui Zhang, Zixin Hu. Writing: Meng Hao, Hui Zhang, Yi Li. Writing – Review and Editing: Zuyun Liu, Xiaofeng Wang, Li Jin.

## Supporting information

Fig S1

Fig S2

Fig S3

Fig S4

Fig S5

Table S1

Table S2

Table S3

Table S4

## Data Availability

The datasets generated during and analysed during the current study are available from the corresponding author on reasonable request.
