# Supplementary figures and images for: Phenotype correlations reveal the relationships of physiological systems underlying human ageing
Source: Aging Cell. 2021 Nov 26;20(12):e13519. doi: 10.1111/acel.13519 (PMC8672793; doi:10.1111/acel.13519)

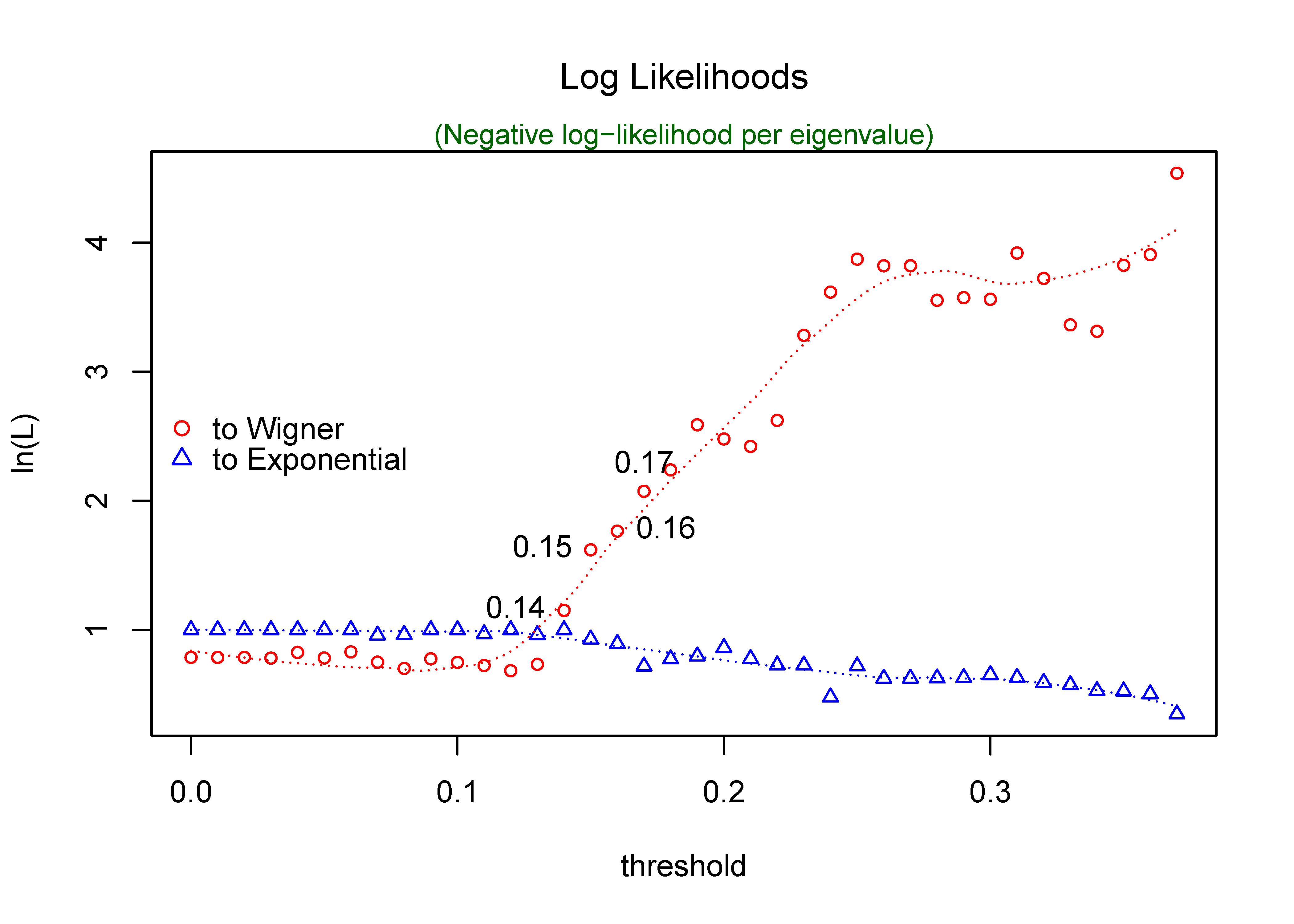

Supplement: Supplementary file 1 — Fig S1 [file ACEL-20-e13519-s004.tif]

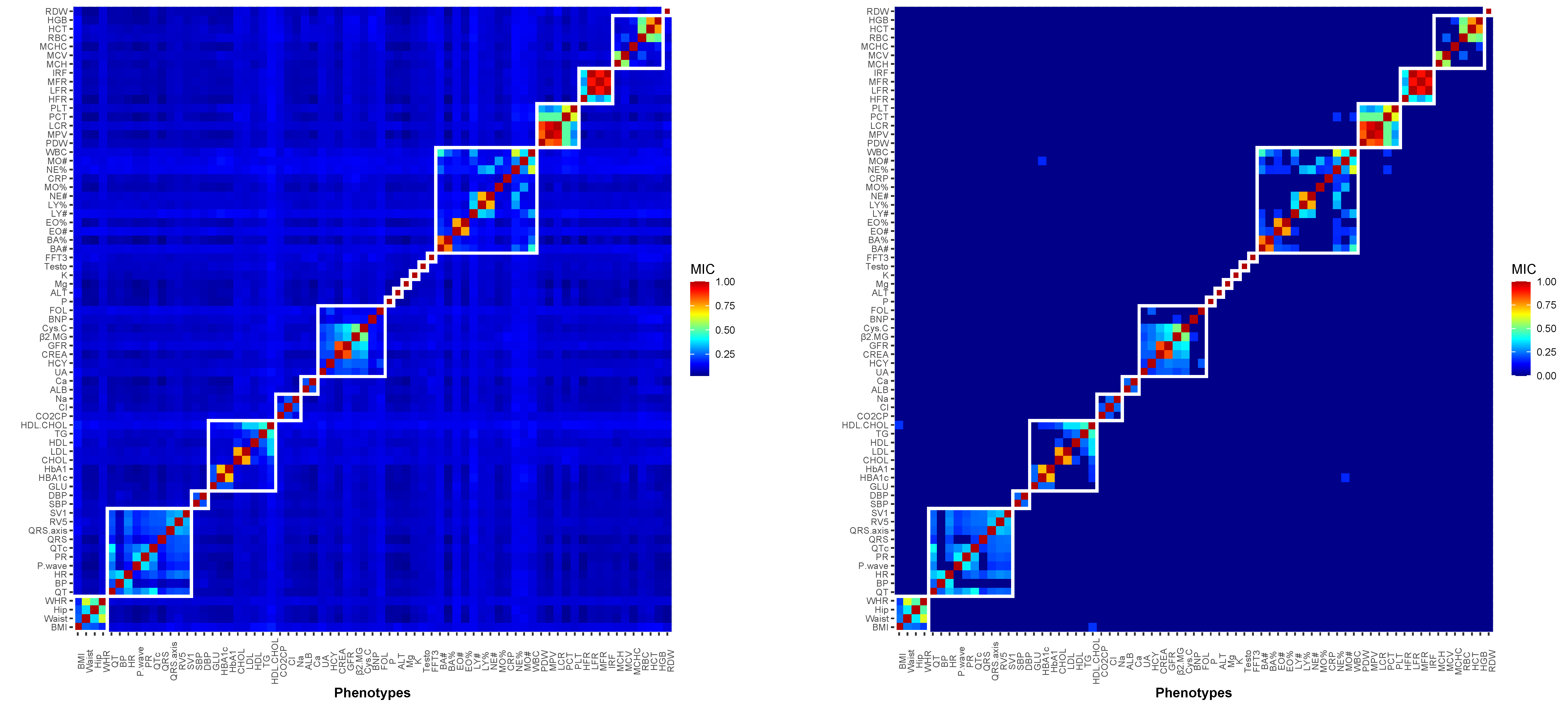

Supplement: Supplementary file 2 — Fig S2 [file ACEL-20-e13519-s008.tif]

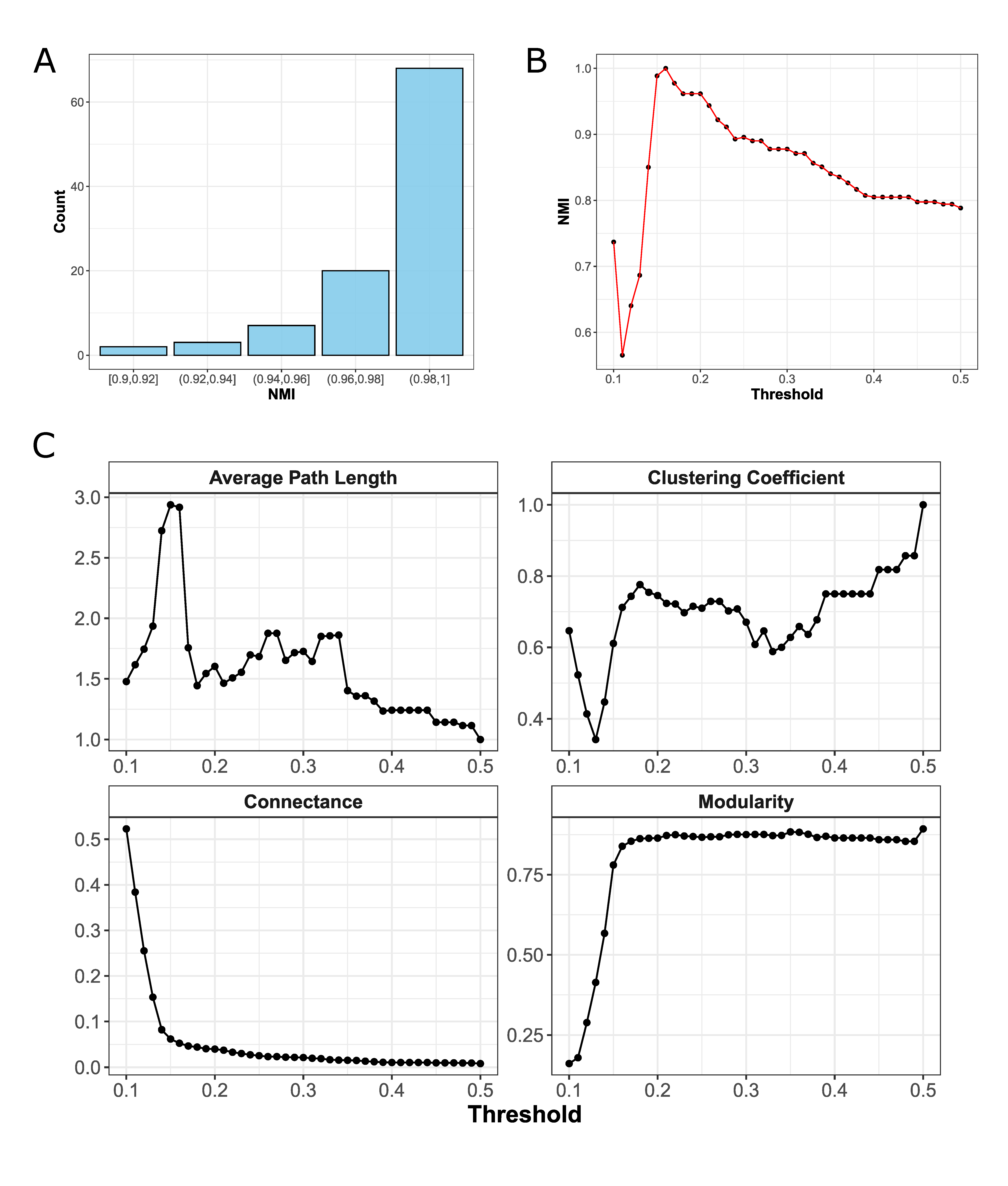

Supplement: Supplementary file 3 — Fig S3 [file ACEL-20-e13519-s001.tif]

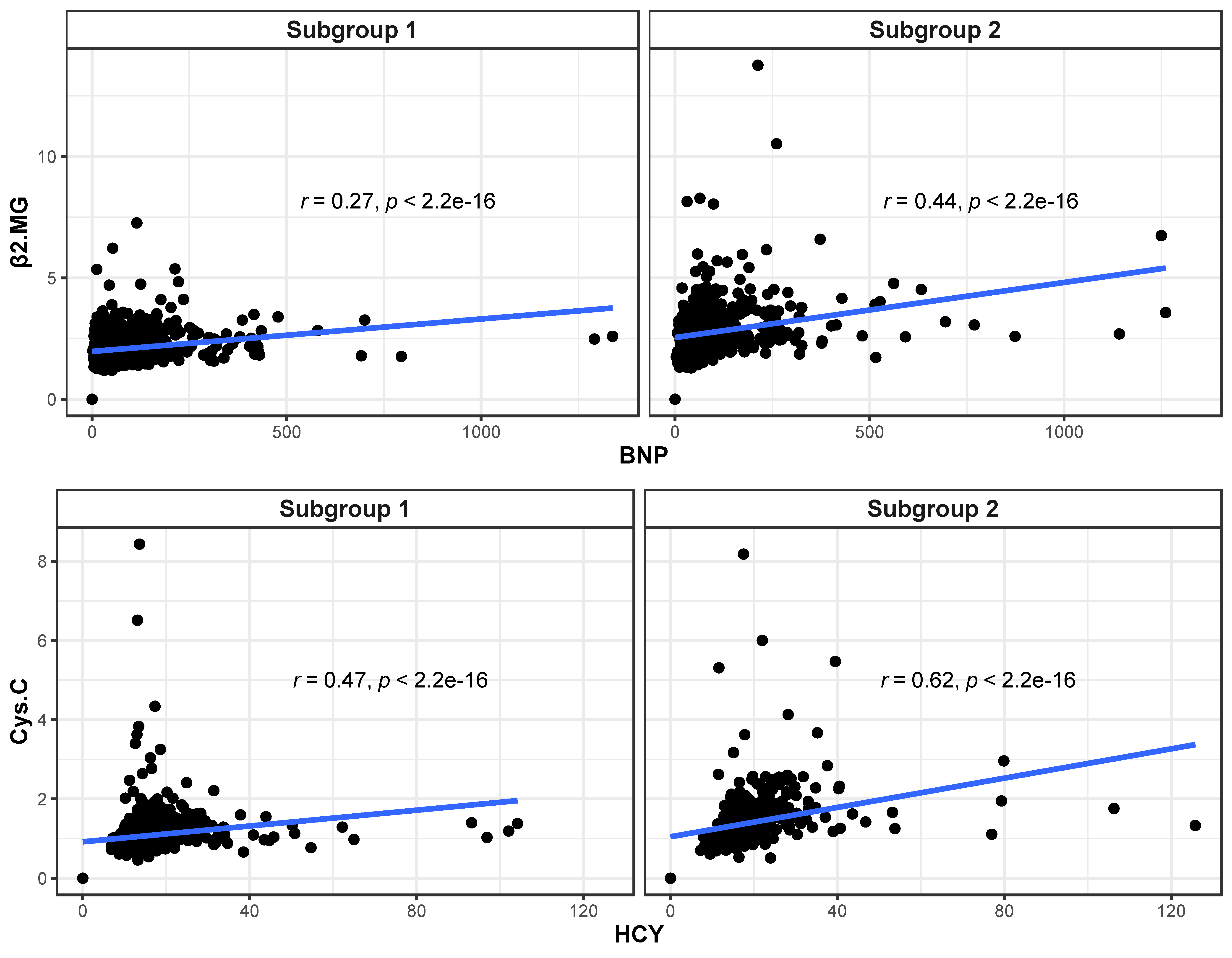

Supplement: Supplementary file 4 — Fig S4 [file ACEL-20-e13519-s007.tif]

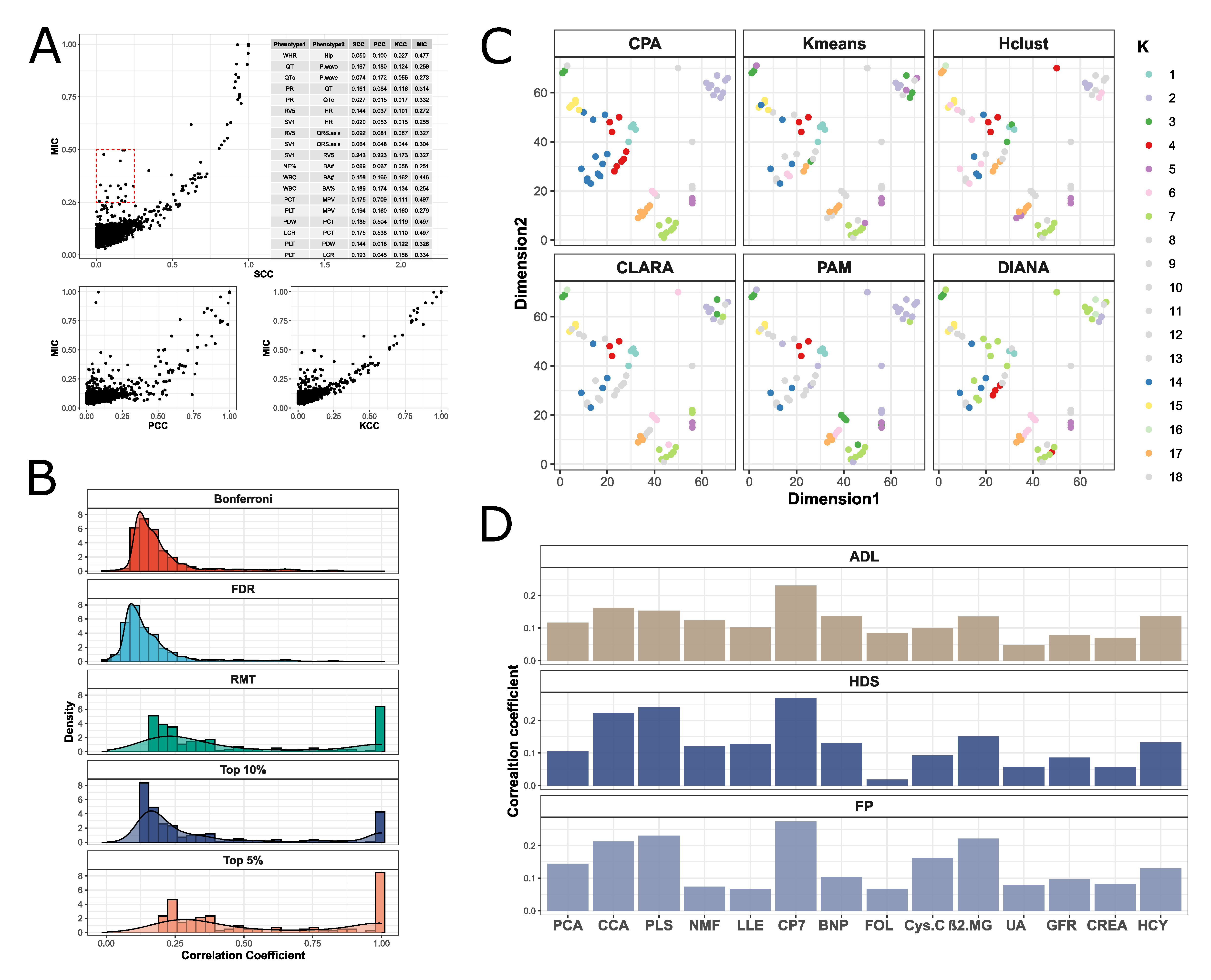

Supplement: Supplementary file 5 — Fig S5 [file ACEL-20-e13519-s009.tif]
