# Supplementary material for: Phenotype correlations reveal the relationships of physiological systems underlying human ageing
Source: Aging Cell. 2021 Nov 26;20(12):e13519. doi: 10.1111/acel.13519 (PMC8672793; doi:10.1111/acel.13519)
Supplement: Supplementary file 7 — Table S2 [file ACEL-20-e13519-s005.docx]

**Supplemental Table 2. Characteristics of study population.**

| Characteristics | Subgroups | Overall (N=1823) |
| --- | --- | --- |
| Age | M ± SD, years | 78.68 ± 4.79 |
|  | < 80 years, n (%) | 1071 (58.75) |
|  | ≥ 80 years, n (%) | 752 (41.25) |
| Gender ^ǂ^ | Males, n (%) | 799 (43.83) |
|  | Females, n (%) | 976 (53.54) |
| Smoking | Yes, n (%) | 440 (24.79) |
|  | No, n (%) | 1383 (75.86) |
| Alcohol assumption | Yes, n (%) | 642 (35.22) |
|  | No, n (%) | 1181 (64.78) |
| Educational status ^ǂ^ | Illiteracy | 815 (44.71) |
|  | Non-illiteracy | 974 (53.43) |
| Marital status | Married^&^, n (%) | 1136 (62.31) |
|  | Others ^&^, n (%) | 667 (36.59) |
| Cardiovascular disease ^Ŧ^ | Yes, n (%) | 215 (11.79) |
|  | No, n (%) | 1608 (88.21) |
| Cancer | Yes, n (%) | 40 (2.19) |
|  | No, n (%) | 1783 (97.81) |
| Chronic kidney disease ^Ŧ^ | Yes, n (%) | 184 (10.09) |
|  | No, n (%) | 1639 (89.91) |
| Anemia | Yes, n (%) | 80 (4.39) |
|  | No, n (%) | 1743 (95.61) |
| Chronic lung disease | Yes, n (%) | 136 (7.46) |
|  | No, n (%) | 1687 (92.54) |
| Cognitive impairment | Yes, n (%) | 909 (49.86) |
|  | No, n (%) | 914 (50.14) |
| Frailty | Yes, n (%) | 322 (17.66) |
|  | No, n (%) | 1501 (82.34) |
| Disability | Yes, n (%) | 169 (9.27) |
|  | No, n (%) | 1654 (90.73) |

^&^ including separated, divorced, never married or widowed. ^ǂ^ Unknown: Sex (48, 2.63%), Educational status (34, 1.87%), marital status (20, 1.10%). ^Ŧ^ Including stroke, myocardial infarction and heart failure. Chronic kidney disease: Participants who had estimated glomerular filtration rate using creatinine.
